# B7-H3 participates in the development of Asthma by augmentation of the inflammatory response independent of TLR2 pathway

**DOI:** 10.1038/srep40398

**Published:** 2017-01-17

**Authors:** Wenjing Gu, Xinxing Zhang, Yongdong Yan, Yuqing Wang, Li Huang, Meijuan Wang, Xuejun Shao, Zhengrong Chen, Wei Ji

**Affiliations:** 1 Department of Respiration, Children’s Hospital Affiliated to Soochow University, Suzhou 215003, China; 2 Department of Clinical laboratory, Children’s Hospital Affiliated to Soochow University, Suzhou 215003, China

## Abstract

B7-H3, a new member of the B7 superfamily, acts as both a T cell costimulator and coinhibitor. Recent studies identified B7-H3 plays a critical role in the development of asthma. But the definitive mechanism is not clear. In this study, we further report that B7-H3 participates in the development of OVA-induced asthma in a murine model. And study its mechanism through the vitro and vivo experiment. Exogenous administration of B7-H3 strongly amplified the inflammatory response and augmented proinflammatory cytokines *in vitro* and *vivo*. These B7-H3–associated proinflammatory effects were not dependent on TLR2 signaling, as airway inflammation, eosinophils infiltration and cytokins (IL-4, IL-5, IL-13 and IFN-gamma) augment were still amplified in TLR2-deficient mice after administrated recombinant mouse B7-H3. These results indicated an important role for B7-H3 in the development of Th1 and Th2 cells in a murine model of asthma and its proinflammatory effects are not dependent on TLR2 signaling.

Asthma is a heterogeneous disease, usually characterized by chronic airway inflammation. It is defined by the history of respiratory symptoms such as wheezing, shortness of breath, chest tightness and cough that vary over time and in intensity, together with variable expiratory airflow limitation^1^. Asthma is the most frequent non-infectious disease in children. Approximately 235 million people worldwide are asthmatics, with an increasing prevalence in low to middle income countries^2^,^3^. Although the rates of hospitalizations and deaths have decreased in recent years, it still caused a heavy burden to the medical and health system.

Asthma is characterized by chronic airway inflammation with massive infiltration of eosinophils, increased mucus production in the bronchioles, and airway hyperreactivity to a variety of specific and nonspecific stimuli^4^. The pathogenesis of asthma is very complex, with immunological factors being the most important. Studies showed that asthmatic patients had an immunity imbalance of Th1/Th2 *in vivo*, with Th2 cells being overactive^5^. The hypersecretion of IL-4, IL-5, IL-13 can promote the production of IgE, which can stimulate the proliferation and activation of eosinophils. The activated eosinophils will secrete a variety of proinflammatory medium and will lead to chronic inflammation of airway^4^,^5^,^6^.

It is well known that B7 family plays a major role in providing a costimulatory or coinhibitory signals for T cells^7^. B7-H3, a newly discovered member of the B7 superfamily, possesses a contrasting role in regulating T cell-mediated immune responses by functioning as both a T cell costimulator and coinhibitor^8^,^9^. Studies show that B7-H3 plays an important role in the development of asthma. Our preliminary research suggested children with asthma exacerbation had significantly higher levels of B7-H3 and blockade of B7-H3 signals markedly reduced allergen-induced asthma in mice^10^,^11^.

Although the definitive counterreceptors for B7-H3 have not yet been identified, previous work revealed that B7-H3 binds to a putative receptor expressed on activated T cells that is distinct from CD28, CTLA-4, ICOS, and programmed death-1^10^,^11^. A recent study shows that B7-H3 strongly augmented both LPS- and bacterial lipoprotein-induced NF-kappaB activation and inflammatory response. This occurred in both a TLR4- and TLR2-dependent manner^12^. Another study shows that B7-H3 participates in the development of experimental pneumococcal meningitis, but this effect is blocked in the TLR2-deficient mice^13^. These B7-H3–associated *in vitro* and *in vivo* effects appeared to be dependent on TLR2 signaling.

However, the relationship between B7-H3 and TLR2 in pathogenisis of asthma has not been fully understood.

The objective of the current study was to explore the relative contribution of B7-H3 to the development of allergic asthma by administering a newly generated recombinant mouse B7-H3 to a mouse model of asthma. In this study, we further identify that B7-H3 substantially augments the airway inflammation, enhances proinflammatory cytokine and chemokine production and aggravates the clinical disease status in a murine model of asthma. And this effect is not blocked in the TLR2-deficient mice.

## Results

### B7-H3 Aggravates Asthmatic Syndromes in Mouse Model

Asthmatic syndromes were observed in mice induced by OVA after aerosol challenge for 30 minutes on days 21 to 27. Many asthmatic syndromes were observed in the wild-type group with asthma, such as cough, tachypnea, dyspnea. And in the recombinant mouse B7-H3 treatment group, the asthmatic syndromes was more obvious, even nodding breathing, abdominal muscle spasm, and stool and urine incontinence came up after 15 minutes’ aerosol challenge. Semi-quantitative scoring showed that the mean (IQR) of the wild-type group with asthma was 1 (1~2), which was significantly higher than that of control group (0 (0~1), *P* = 0.014). In the recombinant mouse B7-H3 treatment group, the semi-quantitative score was 2 (2~2), which was significantly higher than that of the wild-type group with asthma (*P* = 0.019).

### B7-H3 Promotes the Infiltration of Inflammatory Cell in Lung

The recovery ranged from 81% to 98% of the fluid introduced. There was no significant difference among five groups. As shown in Table 1, the wild-type group with asthma showed hypercellularity compared with the normal control group. The total number of cells and percentage of eosinophils and lymphocytes were significantly increased in mice treated with recombinant mouse B7-H3 compared with the control group and the wild-type group with asthma. No significant difference for neutrophils in the BALF was found among three groups.

**Figure 1:**
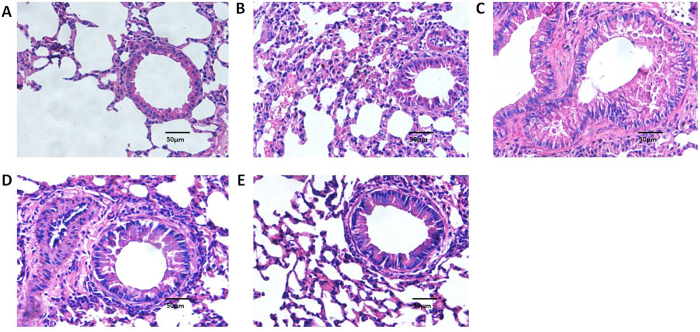
Lung tissue was stained with hematoxylin and eosin (H&E) in various groups (original magnification x400, scale bars = 50 μm). H&E in normal control group (**A**), wild-type group with asthma (**B**), recombinant mouse B7-H3 treatment group (**C**), TLR2-deficient mice group with asthma (**D**), TLR2-deficient mice with recombinant mouse B7-H3 treatment group (**E**).

**Figure 2:**
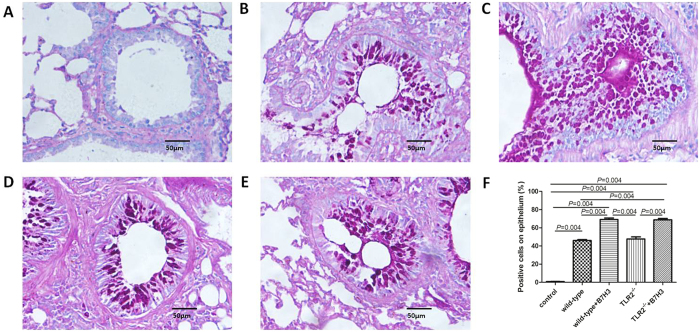
Lung tissue was stained with periodic acid-Schiff (PAS) for mucous secretion in various groups (original magnification x400, scale bars = 50 μm). PAS in normal control group (**A**), wild-type group with asthma (**B**), recombinant mouse B7-H3 treatment group (**C**), TLR2-deficient mice group with asthma (**D**), TLR2-deficient mice with recombinant mouse B7-H3 treatment group (**E**), and values shown are mean ± SD (**F**).

As shown in Fig. 1, administration of recombinant mouse B7-H3 significantly accelerated eosinophil infiltration in lung tissues. The percentage of PAS positive cells on epithelium were 0.77 ± 0.39%, 45.93 ± 2.60%, 69.17 ± 3.94%, 47.70 ± 5.48% and 68.80 ± 3.42% respectively in normal control group, wild-type group with asthma, recombinant mouse B7-H3 treatment group, TLR2-deficient mice group with asthma and TLR2-deficient mice with recombinant mouse B7-H3 treatment group (Fig. 2). Administration of recombinant mouse B7-H3 significantly accelerated mucus overproduction in lung tissues.

### B7-H3 Promotes Th1 and Th2 Cytokine Production in BALF and Plasma

**Figure 3:**
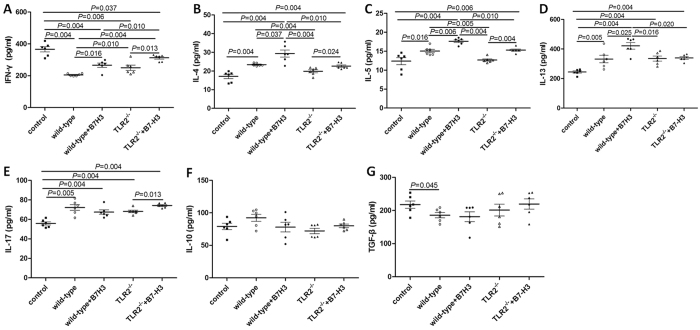
Concentrations of cytokines IFN-gamma (**A**), IL-4 (**B**), IL-5 (**C**), IL-13 (**D**), IL-17 (**E**), IL-10 (**F**) and TGF-beta (**G**) in bronchoalveolar lavage fluid (BALF) in normal control group (control), wild-type group with asthma (wild-type), recombinant mouse B7-H3 treatment group (wild-type + B7-H3), TLR2-deficient mice group with asthma (TLR2^−/−^), TLR2-deficient mice with recombinant mouse B7-H3 treatment group (TLR2^−/−^+B7-H3).

Concentrations of IL-4 (23.40 ± 0.78 pg/mL), IL-5 (15.05 ± 1.13 pg/mL), IL-13 (331.93 ± 61.74 pg/ml), IL-17 (72.21 ± 7.10 pg/ml) in BALF of mice in the wild-type group with asthma were significantly higher than that of control group (*P* < 0.05). Concentration of IFN-gamma (205.12 ± 7.85 pg/ml) and TGF-beta (185.79 ± 19.60 pg/ml) in BALF of mice in the wild-type group with asthma was significantly lower than that of control group (*P* < 0.05). The treatment with recombinant mouse B7-H3 significantly increased the IL-4 (29.34 ± 4.67 pg/mL), IL-5 (17.64 ± 0.84 pg/mL), IL-13 (421.31 ± 53.60 pg/ml) and IFN-gamma (266.01 ± 32.72 pg/ml) concentration in BALF as compared with mice in the wild-type group with asthma (*P* < 0.05). There was no significantly difference of IL-17, IL-10 and TGF-beta in BALF between mice administrated recombinant mouse B7-H3 or not, as shown in Fig. 3.

**Figure 4:**
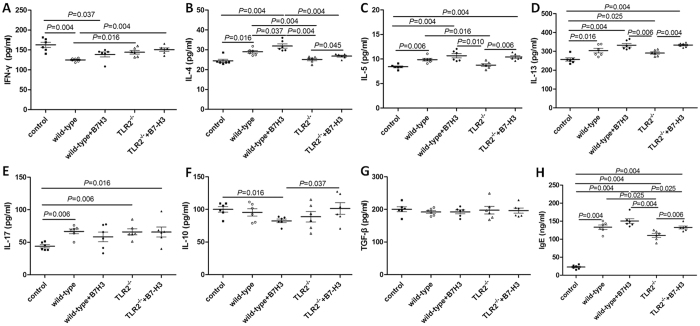
Concentrations of cytokines IFN-gamma (**A**), IL-4 (**B**), IL-5 (**C**), IL-13 (**D**), IL-17 (**E**), IL-10 (**F**), TGF-beta (**G**) and OVA-IgE (**H**) in plasma in normal control group (control), wild-type group with asthma (wild-type), recombinant mouse B7-H3 treatment group (wild-type + B7-H3), TLR2-deficient mice group with asthma (TLR2^−/−^), TLR2-deficient mice with recombinant mouse B7-H3 treatment group (TLR2^−/−^+B7-H3).

Similar phenomenon was also found in plasma. Concentrations of IL-4 (31.88 ± 2.65 pg/mL) in plasma of mice in treated with recombinant mouse B7-H3 were significantly higher as compared with mice in the wild-type group with asthma (*P* = 0.037), as shown in Fig. 4.

### B7-H3 May Promotes OVA-specific immunoglobulin (Ig) E (OVA-IgE) Production

Concentrations of OVA-IgE in plasma of mice in the wild-type group with asthma (133.19 ± 15.05 ng/ml) were significantly higher than that of control group (22.79 ± 5.67 ng/ml) (*P* = 0.004). The treatment with recombinant mouse B7-H3 slightly increased the OVA-IgE (150.30 ± 16.61 pg/mL) concentration in plasma as compared with mice in the wild-type group with asthma, but there was no significantly difference two of them (*P* = 0.109). Data was presented in Fig. 4.

### B7-H3 Promotes Th2 and Th17 Cell Proliferation and Th1, Th2 and Th17 Cytokine Production *in Vitro*

**Figure 5:**
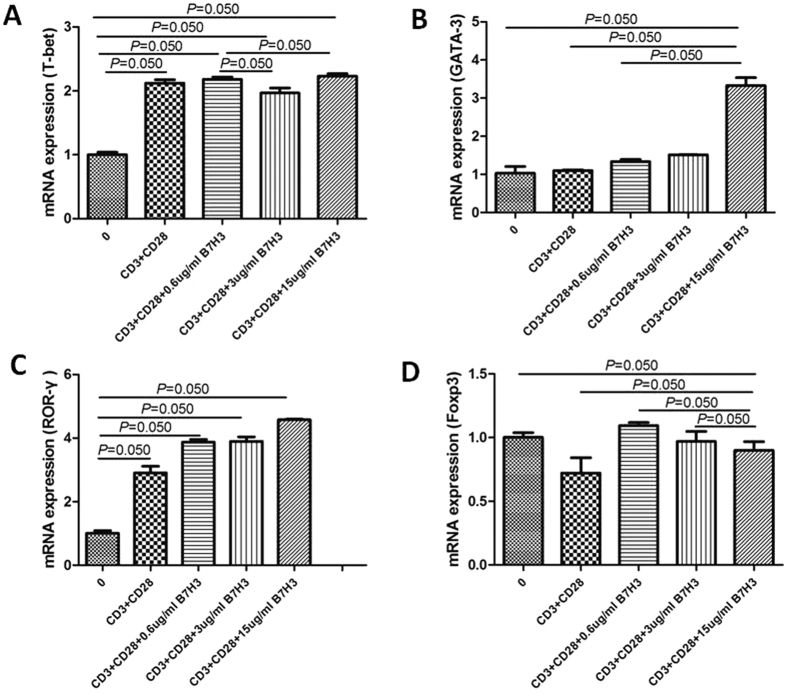
Effect of different dosage of B7-H3 (0 ug/ml, 0.6 ug/ml, 3 ug/ml and 15 ug/ml) on T cell transcription factors T-bet (**A**), GATA-3 (**B**), ROR-γ (**C**) and Foxp3 (**D**) mRNA expression *in vitro*. Quantification was based on fold difference (2^−ΔΔCt^) between groups, using control (0) group as calibrator.

**Figure 6:**
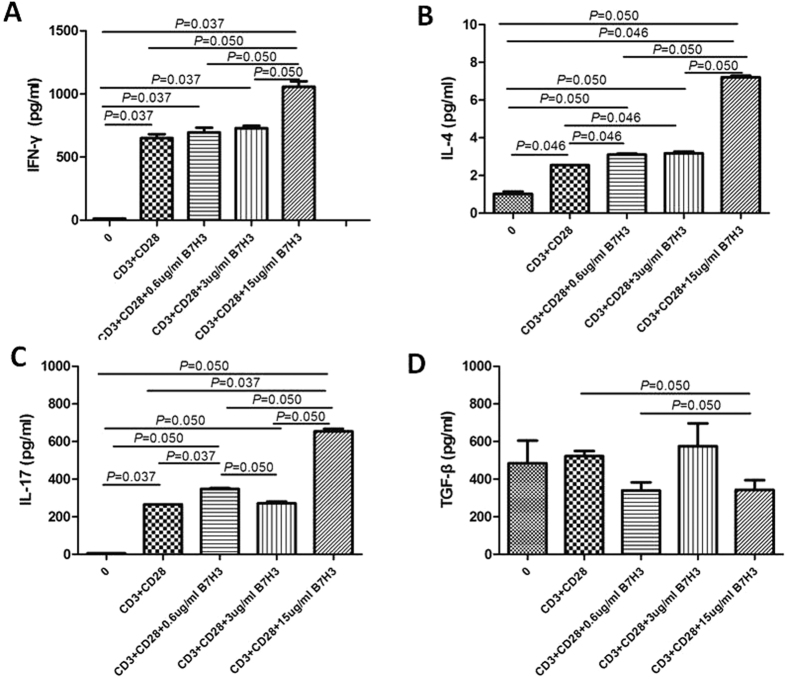
Effect of different dosage of B7-H3 (0 ug/ml, 0.6 ug/ml, 3 ug/ml and 15 ug/ml) on the concentration of cytokines IFN-gamma (**A**), IL-4 (**B**), IL-17 (**C**) and TGF-beta (**D**) in cell-free supernatant *in vitro*.

To evaluate if B7-H3 would affect the proportion of T cell subsets, we carried on the *in vitro* experiment and the local mRNA expression of their respective putative transcription factors was determined. Relative quantification of T-bet, GATA-3, ROR-*γ*, and Foxp3 expression, which are related to Th1, Th2, Th17, and Treg subsets, respectively, was determined. As illustrated in Fig. 5, there was a significant increase in GATA-3 and ROR-*γ* expression with increasing dosage of B7-H3 (P < 0.05). T-bet and Foxp3 expression had no significantly difference among different dosage. On the other hand, IFN-gamma, IL-4 and IL-17 was significantly higher in the cell-free supernatants with high dose (15 ug/ml) of B7-H3 treatment (P < 0.05). But there was no significantly difference of TGF-beta among different groups (Fig. 6).

### TLR2 Deletion Blocks the Development of Asthma

**Figure 7:**
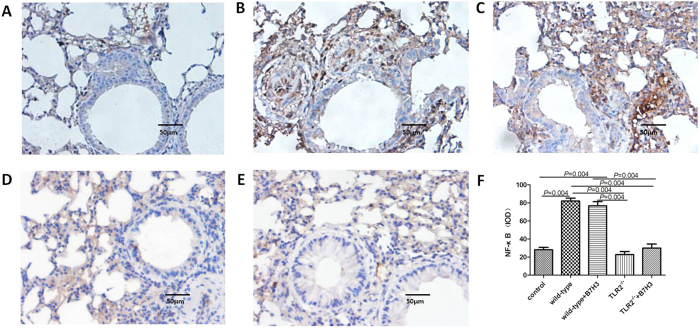
NF-kappaB-positive cells in various groups by immunohistochemistry detection. Cells were counted and statistically analyzed (original magnification x400, scale bars = 50 μm). NF-kappaB expression in normal control group (**A**), wild-type group with asthma (**B**), recombinant mouse B7-H3 treatment group (**C**), TLR2-deficient mice group with asthma (**D**), TLR2-deficient mice with recombinant mouse B7-H3 treatment group (**E**), and values shown are mean ± SD (**F**).

**Figure 8:**
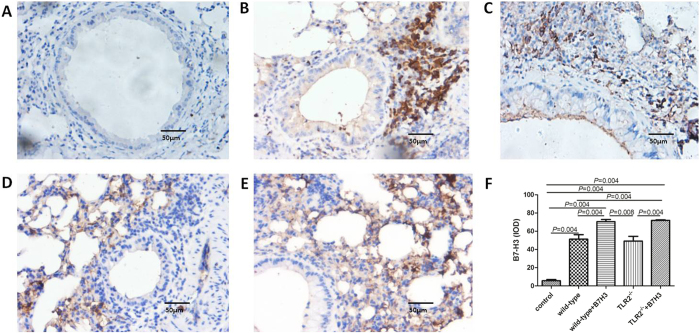
B7-H3-positive cells in various groups by immunohistochemistry detection. Cells were counted and statistically analyzed (original magnification x400, scale bars = 50 μm). B7-H3 expression in normal control group (**A**), wild-type group with asthma (**B**), recombinant mouse B7-H3 treatment group (**C**), TLR2-deficient mice group with asthma (**D**), TLR2-deficient mice with recombinant mouse B7-H3 treatment group (**E**), and values shown are mean ± SD (**F**).

TLR2-deficient mice exhibited significantly milder clinical symptoms (semi-quantitative scoring 1 (0~1)), which was slightly lower than that of the wild-type group with asthma (*P* = 0.056). TLR2-deficient mice exhibited significantly less severe airway inflammation, mucus cell metaplasia, and lower percentage of eosinophils and lymphocytes compared with the wild-type group, as shown in Table 1 and Figs 1 and 2. Meanwhile TLR2 deletion also significantly reduced Th2 cytokine levels compared with the wild-type group. Concentrations of IL-4 (25.09 ± 1.53 pg/ml, 19.85 ± 2.06 pg/mL), IL-5 (8.76 ± 0.70 pg/ml, 12.68 ± 0.74 pg/mL) in plasma and BALF of mice in the TLR2-deficient mice group with asthma were significantly lower than that of wild-type group (*P* < 0.05), and had no significantly difference compared with control group (P > 0.05). Concentrations of OVA-IgE in plasma were also significantly lower in the TLR2-deficient mice group (110.35 ± 13.61 ng/ml) with asthma compared with that of wild-type group (*P* = 0.025). In contrast, concentrations of INF-gamma (144.31 ± 11.24 pg/ml, 250.18 ± 40.88 pg/mL) in plasma and BALF of mice in the TLR2-deficient mice group with asthma were significantly higher than that of wild-type group (*P* = 0.016, 0.010), data was presented in Figs 3 and 4. Immunohistochemical studies showed that NF-kappaB was decreased in TLR2-deficient mouse lungs (Fig. 7). But there was no significantly difference of B7-H3-positive cells in lung between the TLR2-deficient mice group with asthma and the wild-type group (Fig. 8).

### B7-H3 Augments OVA-stimulated Production of Proinflammatory Cytokines and Exacerbates Airway Inflammation Independent of TLR2 Signaling Pathway

After treated with recombinant mouse B7-H3, TLR2-deficient mice exhibited more severe airway inflammation, mucus cell metaplasia, and higher percentage of eosinophils and lymphocytes compared with the TLR2-deficient mice group with asthma, as shown in Table 1 and Figs 1 and 2. Notably, a substantial further accumulation of IL-4 (26.78 ± 1.19 pg/ml, 22.62 ± 1.58 pg/ml), IL-5 (10.41 ± 0.66 pg/ml, 15.30 ± 0.71 pg/ml) and IFN-gamma (150.82 ± 10.51 pg/ml, 312.62 ± 20.39 pg/ml) in plasma and BALF and OVA-IgE (132.58 ± 9.85 ng/ml) in plasma was evident in TLR2-deficient mice challenged with a combination of OVA and B7-H3 compared with the TLR2-deficient mice group with asthma (*P* < 0.05), data was presented in Figs 3 and 4. Immunohistochemical studies showed that B7-H3 was increased in the TLR2-deficient mice with recombinant mouse B7-H3 treatment group (Fig. 8). But there was no significantly difference of NF-kappaB-positive cells in lung between the two TLR2-deficient mice groups (Fig. 7).

## Discussion

Asthma is a chronic airway inflammatory disease with complex pathogenesis, which is closely related to hereditary factor, environmental factor and organism immunity factor^14^,^15^,^16^. Classical immune theories show that the imbalance of Th1/Th2 cells in organism plays an important role in the development of asthma^17^,^18^. Studies show that B7-H3, a newly discovered member of the B7 superfamily, acts as both a T cell costimulator and coinhibitor, and thus plays an important role in regulating T cell-mediated immune responses^8^,^19^. To explore the contribution of B7-H3 to the development of allergic asthma and its mechanism of action, asthma was successfully induced by OVA sensitization and challenge in female C57BL or TLR2-deficient mice. Naïve CD4^+^ T cells were cultured *in vitro* and different dosage of B7-H3 was used for treatment. In this study, we found that the exogenous administration of B7-H3 via intraperitoneal injection strongly amplified the OVA-induced inflammatory response as demonstrated by a substantial further accumulation of proinflammatory cytokines IL-4, IL-5, IL-13 and IFN-gamma in the lung. Vitro experiment showed B7-H3 augment the release of Th1, Th2 and Th17 cytokines. TLR2 deletion blocks the development of asthma, but cannot block the proinflammatory effect caused by B7-H3. Taken together, the treatment with recombinant B7-H3 showed a substantial promotion of the OVA-induced asthmatic responses through proliferating both Th1 and Th2 cells. But, the B7-H3–mediated effects observed in the present study are independent of TLR2 signaling, at least, not only depends on the TLR2 signaling.

Studies show that B7-H3 plays an important role in the development of asthma. Nagashima, *et al*.^4^ found that administration of anti-B7-H3 mAb significantly reduced airway hyperreactivity and decreased production of Th2 cytokines (IL-4, IL-5, and IL-13) in the draining lymph node cells in an asthma model, which indicated B7-H3 may play a critical role in the development of pathogenic Th2 cells. Suh, *et al*.^20^ found that B7-H3-deficient mice developed more severe airway inflammation than did wild-type mice in conditions in which T helper cells differentiated toward Th1 rather than Th2, which suggested B7-H3 was a negative regulator that preferentially affects Th1 responses. Our preliminary research suggested children with asthma exacerbation had significantly higher levels of B7-H3 and blockade of B7-H3 signals markedly reduced allergen-induced asthma in mice^10^,^11^. But a recent study showed that B7-H3 deficient mice developed severe OVA-induced asthma with characteristic infiltrations of eosinophils in the lung, increased IL-5 and IL-13 in lavage fluid, which suggests B7-H3 has a coinhibitory function on Th2 responses^21^. In this study we find that not only the concentration of OVA-IgE, IL-4, IL-5 and IL-13 which are typical markers of the Th2 immune response but also INF-gamma produced by Th1 cells in OVA-induced asthma mice were increased by administration of recombinant B7-H3, which indicated that B7-H3 may play an important role in both Th1 and Th2 cell differentiation and proliferation. Since the receptor of B7-H3 is still unclear, it is hard to judge whether B7-H3 functions by blocking the function of endogenous B7-H3, or by agonizing or activating the putative receptor of B7-H3. But our vitro experiment made up for it. *In vitro* experiment, recombinant mouse B7-H3 direct effects on naïve CD4^+^ T cells and we also found B7-H3 promotes Th2 cell proliferation and Th1 and Th2 cytokine production. Th17 cell also proliferated and IL-17 was elevated when treated with B7-H3 *in vitro*. So to summarize our experiments *in vivo* and *in vitro*, we consider recombinant B7-H3 functions by agonizing or activating the putative receptor of B7-H3. And we conclude that B7-H3 facilitates Th1 and Th2 response.

The definitive costimulatory receptors on monocytes/macrophages responsible for B7-H3–induced augmentation of inflammatory responses have not yet been identified. But it was indicated that B7-H3 binds to a putative receptor expressed on PHA- or anti-CD3 mAb-activated T cells that is distinct from CD28, CTLA-4, ICOS, and programmed death-1^8^,^9^. Hashiguchi, *et al*.^22^ found that TLT-2 might be the receptors of mouse B7-H3, but was quickly denied by Leitner, *et al*.^23^. In a recent study, Zhang, *et al*.^12^ found that B7-H3 amplifies endotoxin/LPS and BLP-stimulated NF-kappaB activation and proinflammatory cytokine production in monocytes/macrophages, which occurs via both TLR4- and TLR2-dependent mechanisms. Chen, *et al*.^13^ also found that B7-H3 was unable to enhance proinflammatory cytokine TNF-alpha, IL-1beta, IL-6, and chemokine MCP-1 release from S. pneumoniae-stimulated TLR2-deficient microglial cells and to upregulate phosphorylated NF-kappaB p65 and MAPK p38 expression in S. pneumoniae-stimulated TLR2-deficient microglial cells. These studies demonstrated that in infection models, B7-H3 amplified the inflammatory response through a TLR2-dependent mechanism. In this study, TLR2-deficient mice were sensitized and chanllenged to induce asthma with OVA, and recombinant mouse B7-H3 was administrated in one group. We found that TLR2-deficient mice exhibited significantly less severe airway inflammation, mucus cell metaplasia, and lower percentage of eosinophils compared with the wild-type mice. They also had lower levels of IL-4, IL-5 and a higher level of IFN-gamma in BALF compared with the wild-type mice, which suggested OVA-induced experimental allergic asthma is TLR2 dependent and TLR2 deletion can block the development of asthma. However, like their wild-type littermates, after treated with recombinant mouse B7-H3, TLR2-deficient mice exhibited more severe airway inflammation, mucus cell metaplasia, and higher percentage of eosinophils compared with those not treated with B7-H3. OVA-IgE, IL-4, IL-5, IL-13 and INF-gamma in BALF was also increased in TLR2-deficient mice treated with B7-H3, which suggest in OVA-induced asthma model B7-H3 augment the inflammatory response independent of TLR2 pathway. We conjecture several pathways may participate in the proinflammatory response caused by B7-H3, and TLR-2 signaling is only one of them.

Review of literature, we found the studies for B7-H3 were focus on the tumor. Zhang T, *et al*.^24^ found that B7-H3 induce Bcl-2 and Bcl-xl overexpression via the Jak2-STAT3 signaling pathway to inhibit cancer cell apoptosis. Xie C, *et al*.^25^ found that soluble B7-H3 promotes the invasion and metastasis of pancreatic carcinoma cells through the TLR4/NF-κB pathway. And Jiang B, *et al*.^26^ found B7-H3 increases thymidylate synthase expression via the PI3k-Akt pathway. In OVA-induced asthma model, other mechanisms may participate in the inflammatory response caused by B7-H3, which need further research and maybe we can finding a breakthrough from them.

In conclusion, our data suggest that a substantial exacerbation of the OVA-induced asthmatic responses is observed by administration of recombinant B7-H3 through augmenting both Th1 and Th2 characterized cytokines. TLR2 deletion blocks the development of asthma, but cannot block the proinflammatory effect caused by B7-H3, which suggest the B7-H3–mediated effects observed in the present study are independent of TLR2 signaling, at least, not only depends on the TLR2 signaling.

## Methods

### Mice and recombinant mouse B7-H3

Female, specific-pathogen-free, C57BL mice (6–8 weeks old and weighing 18–20 g) and TLR2-deficient mice on the C3H background were purchased from the Animal Use Center of Soochow University, Suzhou, China. Mice were housed in barrier cages under controlled environmental conditions (12/12 h light/dark cycle, 55 ± 5% humidity, 23 ± 1°C). They had free access to pelleted food and tap water. The animal experiments were performed according to the European Community guidelines for care and use of animals and approved by the Ethics Committee for Animal Use of Soochow University. The recombinant mouse B7-H3 was purchased from R&D Systems, Minneapolis, America.

### Experimental groups

Wild-type and TLR2-deficient mice were randomized into one of the following five experimental groups: the normal control group, the wild-type group with asthma, the recombinant mouse B7-H3 treatment group, the TLR2-deficient mice group with asthma, and the TLR2-deficient mice with recombinant mouse B7-H3 treatment group. For the wild-type group with asthma, the recombinant mouse B7-H3 treatment group, the TLR2-deficient mice group with asthma, and the TLR2-deficient mice with recombinant mouse B7-H3 treatment group, 0.5 ml ovalbumin (OVA) suspension (Sigma-Aldrich, Los Angeles, California), which contained 100 μg OVA and 400 μg aluminium hydroxide gel in phosphate-buffered saline (PBS), was intraperitoneally administered on days 0, 7and 10. From the 21^st^ day after the injection, the animals were challenged with aerosolized 1% OVA daily for 7 consecutive days. For the recombinant mouse B7-H3 treatment group and the TLR2-deficient mice with recombinant mouse B7-H3 treatment group, 0.5 ml of 100 μg/ml recombinant mouse B7-H3 was administered intraperitoneally on days 0, 3, 7, 10, 14 after the first OVA injection. The normal control group mice were administered PBS intraperitoneally on the same days and inhaled PBS in a similar manner. The performance of mice was recorded and we graded each of them according to their severity (0: no obvious breathing symptom; 1: mild symptom such as cough, tachypnea, mild dyspnea; 2: severe symptom such as nodding breathing, abdominal muscle spasm, and stool and urine incontinence).

### Specimen collection

24 hours after the last inhalation of OVA, the mice were killed after anesthesia. Blood was drawing-out from their hearts and anticoagulated with EDTA. Right lung was ligated and removed for histological analysis. The left principal bronchus was cannulated with a polyethylene tube through which the lungs were gently lavaged 3 times with 0.5 ml PBS containing 10% fetal calf serum. A total of 1.5 ml bronchoalveolar lavage fluid (BALF) was collected.

### Cytology in Bronchoalveolar Lavage

The concentration of cells in the BALF was determined by cytometry. The total number of cells in the BALF was then calculated. BALF was then centrifuged at 300 g for 10 minutes, and sediment cell smears were stained with Wright-Gimsa mixed coloring method for cell categorization (Wright-Giemsa Stain, Baso Diagnostics Inc, Taiwan, China). At least 500 cells were examined for each specimen. The ratios of various cell types in total cell counts were reported. The supernatant was stored at −20 °C for cytokine detection.

### Histological Analysis of Lung Sections

The right lungs were first perfused with 4% paraformaldehyde in 0.1 M PBS, fixed with formalin, and embedded with paraffin. Paraffin sections (3 μm) were stained with hematoxylin and eosin (H&E) and periodic acid-Schiff (PAS) as measurements of eosinophil infiltration and mucus production, respectively. We used a point counting method to quantify the percentage of cells stained positively by dividing the total number of epithelial cells counterstained with hematoxylin in five random fields from each of the subjects. PAS-positive cell count was based on counting nucleus to the nearest apical surface immediately beneath the PAS stain; whereas ciliated cells were determined by counting nucleus nearest to the apical surface in cells with cilia but without PAS staining. Other paraffin sections were incubated with the rat anti-B7-H3 mAb or anti-NF-kappaB (p65) mAb at 4 °C overnight after antigen repair. After incubation with anti-rat IgG antibody conjugated with horseradish peroxidase (KPL, London, England), the specimens were developed using the 3,30-diaminobenzidine coloration method. Ten sections were randomly selected and analyzed by using Image-Pro Plus 6.0 software. B7-H3- or NF-kappaB-positive cells per square millimeter were quantified.

### *In vitro* T cell proliferation assay

Human naïve CD4^+^ T cells isolated from healthy human (donate from Red Cross) were diluted into 5 × 10^5^ cells/ml and seeded into 96-well flat-plate that was pre-coated with anti-CD3 mAb (50 ng/ml) and anti-CD28 mAb (500 ng/L). Then, a different dose of B7-H3 (0 ug/ml, 0.6 ug/ml, 3 ug/ml and 15 ug/ml) was also added into the wells. After a 96 h culture at 37 °C, 5% CO_2_, proliferation of T cells was harvested on a Micro 96 Harvester. Cell-free supernatants were collected to measure cytokines and cells were collected to measure the relative expression of mRNA. Naïve CD4^+^ T cells without stimulus were used for control.

### ELISA for cytokines

Blood was centrifuged at 300 g for 10 min and plasma was collected. The concentrations of proinflammatory cytokines IL-4, IL-5, IL-10, IL-13, IL-17, TGF-beta, IFN-gamma and OVA-IgE in plasma and BALF were assessed by ELISA (R&D Systems, Minneapolis, America) according to the manufacturer’s instructions.

Cell-free supernatants from T cell proliferation assay *in vitro* were assayed for IL-4, IL-17, IFN-gamma and TGF-beta by ELISA (R&D Systems, Minneapolis, America) according to the manufacturer’s instructions.

### Local mRNA Expression for T cell Subsets Transcription Factors

Cells from T cell proliferation assay *in vitro* were collected, mRNA was extracted using TRIzol (Life Technologies) and RNeasy Mini Kit (Qiagen, Valencia, CA, USA). DNase treatment followed manufacturer’s instructions and purity of the samples was assessed by 260/280 ratio. Singlestrand cDNA synthesis was performed from 100 ng/*μ*L of extracted RNA, using the High Capacity cDNA Reverse Transcription Kit (Applied Biosystems, Carlsbad, CA, USA). Aliquots of cDNA (3 *μ*L) were subjected to real time PCR reaction using TaqMan system. Each reaction contained 15 *μ*L of TaqMan Gene Expression Master mix (Life Technologies, Carlsbad, CA, USA), 0.25 *μ*L of the reference gene, and 1.0 to 1.5 *μ*L of the target genes. The following inventoried primes/probes tested by Life Technologies were used: T-bet (Mm00450960_m1), GATA-3 (Mm00484683_m1), ROR-*γ* (Mm01261022_m1), and Foxp3 (Mm00475162_m1). The reactions were performed in ABI 7300 equipment (Applied Biosystems, Carlsbad, CA, USA) using standard parameters. Data were analyzed in SDS Software System 7300 and relative quantification was determined based on fold difference (2^−ΔΔCt^) using Ct value of the target gene normalized to the reference gene and the control (0) group as the calibrator.

### Statistical Analysis

All data are expressed as mean ± standard deviation (SD). Statistical analysis was performed by SPSS 18.0 software (SPSS Inc., Chicago, IL), using the rank sun test and one-way analysis of variance. P < 0.05 was considered statistically significant.

## Additional Information

**How to cite this article**: Gu, W. *et al*. B7-H3 participates in the development of Asthma by augmentation of the inflammatory response independent of TLR2 pathway. *Sci. Rep.* **7**, 40398; doi: 10.1038/srep40398 (2017).

**Publisher's note:** Springer Nature remains neutral with regard to jurisdictional claims in published maps and institutional affiliations.

## Figures and Tables

**Table 1:** Cellular profile in bronchoalveolar lavage fluid in different groups (n = 6).

	Recovery (%)	Total cells (×10^4^/ml)	Eosinophils (%)	Neutrophils (%)	Lymphocytes (%)	Macrophagocyte (%)
Control group	87.33 ± 7.29	198.00 ± 15.21^*^	1.37 ± 0.49^*^	1.78 ± 0.38	16.00 ± 1.24^*^	80.85 ± 1.67^*^
Wild-type group	87.00 ± 4.60	303.33 ± 18.07^#^	14.55 ± 0.60^*^	2.38 ± 0.42	25.60 ± 4.30^#^	57.47 ± 4.52^*^
Wild-type + B7H3 group	88.17 ± 5.91	356.50 ± 16.67^*^	17.18 ± 1.26^*^	1.88 ± 0.29	31.87 ± 2.06^*^	49.07 ± 3.24^*^
TLR2^−/−^ group	94.50 ± 2.07	226.50 ± 20.36^*^	3.51 ± 1.28^*^	1.90 ± 0.44	16.38 ± 1.69*	78.20 ± 1.79^*^
TLR2^−/−^ + B7-H3 group	90.67 ± 3.50	288.00 ± 22.54^#^	7.67 ± 0.95^*^	3.00 ± 0.37^*^	22.98 ± 1.89^#^	66.35 ± 2.84^*^
χ^2^	7.310	25.766	27.504	16.679	24.671	26.875
*P*	0.120	<0.001	<0.001	0.002	<0.001	<0.001

Control group, the normal control group; wild-type group, the wild-type group with asthma; wild-type + B7-H3 group, the recombinant mouse B7-H3 treatment group; TLR2^−/−^ group, the TLR2-deficient mice group with asthma; TLR2^−/−^ +B7-H3 group, the TLR2-deficient mice with recombinant mouse B7-H3 treatment group. *<0.05 compared with other group. ^#^<0.05 compared with C group, WB group and T group.
